# A dataset on tail risk of commodities markets

**DOI:** 10.1016/j.dib.2017.09.005

**Published:** 2017-09-11

**Authors:** Robert J. Powell, Duc H. Vo, Thach N. Pham, Abhay K. Singh

**Affiliations:** a Edith Cowan University, Australia; b Ho Chi Minh City Open University, Vietnam

## Abstract

This article contains the datasets related to the research article “The long and short of commodity tails and their relationship to Asian equity markets”(Powell et al., 2017) [1]. The datasets contain the daily prices (and price movements) of 24 different commodities decomposed from the S&P GSCI index and the daily prices (and price movements) of three share market indices including World, Asia, and South East Asia for the period 2004–2015. Then, the dataset is divided into annual periods, showing the worst 5% of price movements for each year. The datasets are convenient to examine the tail risk of different commodities as measured by Conditional Value at Risk (CVaR) as well as their changes over periods. The datasets can also be used to investigate the association between commodity markets and share markets.

**Specifications table**

**Table:** 

Subject area	*Finance & Economics*
More specific subject area	*Financial risk, Tail risk*
Type of data	*Table, graph, R code, EViews Code, Excel file*
How data was acquired	*The datasets were obtained from Datastream*
Data format	*Raw, estimated*
Experimental factors	*The dataset is divided into annual periods showing the tail 5% price movements for each year.*
Experimental features	*Secondary data of price indices for commodities markets and share markets*
Data source location	*Datastream*
Data accessibility	*Data is with this article*

.

**Value of the data**

• The data are convenient to examine the tail risk of different commodities, using Conditional Value at Risk (CVaR), or other tail risk measures.
• The data can be used to examine the changes in relative tail risk over the period 2004–2015.
• The data, together with the codes, can be used to undertake Vector Autoregression (VAR) analysis to explore the spillovers between commodities and between commodities and share markets.
• The dataset is useful for different types of analysis in areas such as cointegration estimation between different commodities.

## 1 Data

The raw data, which is reported in the “Data.xlsx” excel file, includes the daily price index of 24 different commodities indices decomposed from S&P GSCI index, divided into 5 sub-categories (i.e. agriculture, energy, livestock, industrial metals, and precious metals). The 24 commodities making up the sub-categories are shown in Table 1 together with descriptive statistics of the daily price movements. The daily price movement data are also shown in the excel file, together with an annual breakdown of the worst 5% of price movements for each commodity for the period from January 01 2004 to December 31 2015. Note that the raw data contains information prior to 2004 in order to provide historical data for the rolling window approach we mention below.

**Table 1:** Daily commodity market price movements (2004–2015).

Index	Max (%)	Min (%)	Average (%)	S.D (%)
**Total market**	7.21	−8.45	0.02	1.50
*Energy*	9.81	−9.35	0.00	1.92
Crude Oil	13.34	−13.07	0.00	2.18
Brent Crude	12.88	−10.48	0.01	2.01
Unleaded Gasoline	12.97	−11.18	0.01	2.26
Heating Oil	9.91	−9.68	0.01	1.98
Gas Oil	10.73	−8.50	0.01	1.80
Natural Gas	17.13	−13.80	−0.03	2.89
*Industrial Metals*	7.58	−9.11	0.01	1.59
Aluminium	5.93	−8.26	0.00	1.46
Copper	11.90	−10.38	0.02	1.87
Lead	12.84	−13.03	0.03	2.23
Nickel	13.16	−18.22	−0.02	2.44
Zinc	9.93	−11.13	0.01	2.06
*Precious Metals*	8.76	−10.11	0.03	1.29
Gold	8.59	−9.81	0.03	1.21
Silver	12.47	−19.49	0.03	2.20
*Agriculture*	7.15	−7.63	0.01	1.37
Wheat	8.79	−9.97	0.01	2.04
Wheat (Kans)	8.10	−8.99	0.01	1.85
Corn	8.66	−8.12	0.01	1.86
Soybeans	6.43	−7.34	0.00	1.62
Cotton	6.94	−7.13	−0.01	1.74
Sugar	8.18	−12.37	0.03	2.02
Coffee	12.06	−11.25	0.02	1.99
Cocoa	8.99	−9.78	0.02	1.75
*Livestock*	3.25	−3.57	0.02	0.88
Feeder Cattle	4.25	−3.62	0.02	0.90
Live Cattle	3.70	−3.34	0.02	0.92
Lean Hogs	7.35	−6.62	0.00	1.51

**Fig. 1:**
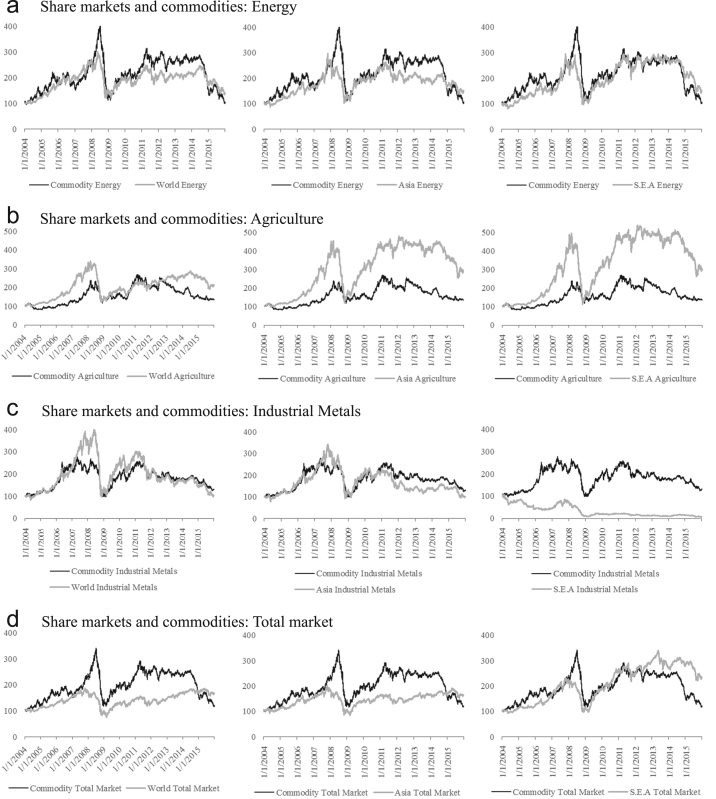
a. Share markets and commodities: energy, b. Share markets and commodities: agriculture, c. Share markets and commodities: industrial metals, d. Share markets and commodities: total market.

Moreover, share market data of three main commodity categories (agriculture, industrial metals and energy) at three regional levels, including Global, Asia, and South East Asia, are also collected (see Table 2). The movements of commodity markets and different share markets is shown in Fig. 1a – d.

**Table 2:** Daily share market price movements (2004–2015).

Index	Max (%)	Min (%)	Average (%)	S.D (%)
**World share market**				
Total market	8.17	−6.67	0.02	0.99
Energy	10.09	−11.88	0.01	1.40
Agriculture	10.41	−8.40	0.02	1.10
Industrial Metals	9.94	−13.00	0.00	1.62
				
**Asia share market**				
Total market	8.85	−7.56	0.02	1.11
Energy	8.12	−10.46	0.01	1.40
Agriculture	11.28	−9.27	0.03	1.25
Industrial Metals	11.01	−11.46	0.00	1.62
			
**South East Asia share market (S.E.A)**				
Total market	7.03	−6.64	0.03	1.01
Energy	10.92	−15.39	0.01	1.59
Agriculture	12.38	−11.14	0.04	1.35
Industrial Metals	15.16	−28.24	−0.09	2.32

## 2 Experimental design, materials and methods

The datasets, including the daily price of 24 indices from commodity markets and 9 indices from share markets, are collected from Datastream from which the daily price movements are calculated.

We [1] use the data to examine the relative tail risk of commodities, using Conditional Value at Risk (CVaR). This CVaR is calculated for each commodity for each year in the sample, to investigate whether there is a change in relative tail risk over time. We then group them into periods of equal length of 3 years each representing pre-GFC (2004–2006), GFC (2007–2009), post-GFC (2010–2012) and recent (2013–2015) to examine changes in CVaR over these periods.

The GSCI index is generally regarded as providing investors with a reliable and publicly available benchmark for investment performance in the commodity markets. It should be noted that GSCI provide a spot index, a total return index and an excess return index which provide different price movement results due to futures investment features such as roll factors. However, on a volatility basis (standard deviation, Value at Risk or CVaR) we find no significant differences between the three indices in any of our examined periods. As our focus in this study is based on tail volatility in prices (CVaR), we find that all three indices will achieve significantly similar tail risk results.

To assess whether there is any significant association in CVaR between commodities and their corresponding share market category in each region (Global, Asia, and South East Asia), a rolling GARCH model, a bivariate Vector Autoregression model, and Granger Causality tests are applied.

Firstly, the most appropriate GARCH (p, q) model is obtained for each index using R software. The code is given in Supplementary material (“RCode.txt”).

Next, we use the above GARCH (p, q) to estimate rolling window models with the window size of 2 years (520 observations based on trading days) and forecast the daily Value at Risk (VaR) using EViews software. The code is given in Supplementary material (“EViewsCode.txt”). We then calculate CVaR, which is based on the price movements beyond VaR as outlined in the article.

Finally, a bivariate Vector Autoregression model is fitted for each of the 12 pairs comprising each share market and its corresponding commodity group (3 regions × 4 indices, i.e. total market, agriculture, energy, and industrial metals).

## References

[1] R.J. Powell, D.H. Vo, T.N. Pham, A.K. Singh, The long and short of commodity tails and their relationship to Asian equity markets. Asian Econ. 52 (2017) 32-44.

